# Effects of Whey Protein Isolate-Based Film Incorporated with Tarragon Essential Oil on the Quality and Shelf-Life of Refrigerated Brook Trout

**DOI:** 10.3390/foods10020401

**Published:** 2021-02-11

**Authors:** Maria-Ioana Socaciu, Melinda Fogarasi, Elemér Lajos Simon, Cristina Anamaria Semeniuc, Sonia Ancuţa Socaci, Andersina Simina Podar, Dan Cristian Vodnar

**Affiliations:** 1 Department of Food Science, University of Agricultural Sciences and Veterinary Medicine Cluj-Napoca, 3-5 Mănăştur St., 400372 Cluj-Napoca, Romania; maria-ioana.socaciu@usamvcluj.ro (M.-I.S.); elemer.simon@usamvcluj.ro (E.L.S.); sonia.socaci@usamvcluj.ro (S.A.S.); andersina-simina.podar@usamvcluj.ro (A.S.P.); 2 Department of Food Engineering, University of Agricultural Sciences and Veterinary Medicine Cluj-Napoca, 3-5 Mănăştur St., 400372 Cluj-Napoca, Romania; melinda.fogarasi@usamvcluj.ro

**Keywords:** film, whey protein isolate, tarragon essential oil, brook trout, refrigerated storage, physicochemical quality, microbiological quality, sensory quality, shelf-life

## Abstract

The efficiency of some films prepared from heat-denatured whey protein isolate solutions on the quality and shelf-life of brook trout samples during storage at 4 °C was studied in this research (WPIf-a film based on whey protein isolate and WPIf+2.5%TEO-a film based on whey protein isolate incorporated with 2.5% tarragon essential oil). The control and covered fish samples were periodically assessed (at 3 days) over 15 days of storage for the physicochemical (pH; EC, electrical conductivity; TVB-N, total volatile basic nitrogen; TBARS, thiobarbituric acid reactive substances; color), microbiological (TVC, total viable count; PTC, psychrotrophic count; LAB, lactic acid bacteria; H_2_S-producing bacteria), and sensory properties (color discoloration; odor; overall acceptability). The WPIf+2.5%TEO has proven enhanced quality preservation effects compared to WPIf by showing lower values for physicochemical parameters, lower microbial loads, and higher sensory scores in the fish sample. All these effects have led to an extension of the sample’s shelf-life. In conclusion, the tarragon essential oil has conferred antioxidant and antimicrobial properties to the film. Thus, the WPIf+2.5%TEO could be a promising material for the packaging of fresh brook trout during refrigerated storage.

## 1. Introduction

The brook trout (*Salvelinus fontinalis*) is a species of freshwater fish in the genus *Salvelinus* of the family Salmonidae [1]. Originally found only in north-eastern North America, it was subsequently introduced to western Canada and the United States, as well as South America, New Zealand, Asia, and several regions of Europe [2]. Fish is one of the most commercialized, but also perishable, food products due to high water activity, nutrient availability, nearly neutral-pH, and the presence of autolytic enzymes [3,4]. Under normal refrigerated storage conditions, its shelf-life is limited by the development of enzymatic and chemical reactions [4]. Therefore, keeping fish quality during the supply chain is a challenge for food manufacturers [5].

In recent years there has been an increase in the development of active packaging materials, based on natural polymers, by incorporating essential oils [4,6]. It is well known that essential oils possess antimicrobial and antioxidant properties [7,8]. However, depending on the scent intensity or the level of incorporation, these may impart foreign taste and aroma to food. Thyme and oregano essential oils, the active substances most used in packaging materials, have strong scent intensities. Others, like tarragon essential oil, have a weaker scent.

Previous research has shown that tarragon essential oil (*Artemisia dracunculus* L.) possesses antibacterial, antifungal, and antioxidant properties [9,10]. Russian tarragon and French tarragon are the best known regional “varieties” of *A. dracunculus*. The chemical composition of their essential oils was found to be different; terpinen-4-ol, sabinene, and elemicin are the main components of Russian tarragon and estragole is the predominant compound of French tarragon [11]. The essential oil of tarragon has been successfully used as an active ingredient in the development of a hake protein-based edible film [12], of a chitosan-based coating (for maintaining the postharvest quality of kumquats fruits) [13], and of a chitosan-gelatin coating (for the preservation of pork slices) [14].

Natural polymers, such as protein and polysaccharides, are commonly used to prepare films and coatings for food applications. Several studies have investigated the efficiency of such active packaging materials in retarding fish spoilage during refrigerated storage: a gelatin-alginate film containing oregano essential oil on rainbow trout slices [15], a whey protein concentrate coating incorporated with cinnamon essential oil on beluga sturgeon fillets [16], a coating based on chitosan and gelatin on golden pomfret fillets [17], a whey protein coating incorporated with lactoperoxidase and *α*-tocopherol on pike-perch fillets [18], and a gelatin-chitosan coating incorporated with clove essential oil on tuna fillets [19]. The antimicrobial effect of chitosan has been widely reported, but the acetic acid used to prepare its solution gives the coating a strong acidic flavor [20,21]. Moreover, chitosan is substantially more expensive than other polymeric matrices such as whey protein.

Films based on whey protein are usually obtained by casting and drying of aqueous WPI (whey protein isolate) and WPC (whey protein concentrate) solutions; these films have shown moderate moisture barrier properties but good oxygen barrier properties [22]. In a previous study, we have developed a WPI-based film incorporated with 2.5% tarragon essential oil [6]. This study aimed to investigate the effectiveness of this film in maintaining the quality, thereby extending the shelf-life of refrigerated brook trout. As far as we know, no research work has considered such a case study on brook trout. The packaging materials incorporated with essential oils previously developed were not investigated for their effect on fish sensory properties. For this purpose, physicochemical, microbiological, and sensory properties of brook trout samples were evaluated during 15 days of storage at 4 °C in the current study.

## 2. Materials and Methods

### 2.1. Materials

Live brook trout specimens (*Salvelinus fontinalis*), with an average weight of 650 ± 20 g, were harvested from a local aquaculture farm (Gilău, Cluj County, Romania) in July 2020.

Whey protein isolate (Prolacta 95 LL Instant, Lactalis, France) was purchased from REDIS C.O. S.R. (Bucharest, Romania) and glycerol from Chempur (Piekary Śląskie, Poland). Tarragon essential oil (*Artemisia dracunculus* L.) was purchased from Aroma-Zone (Cabrières, France). n-Hexane for gas chromatography was purchased from Merck KGaA (Darmstadt, Germany). Perchloric acid, antifoam silicone, sodium hydroxide, boric acid, methyl red, methylene blue, and 95% ethanol were purchased from VWR International, LLC (Fontenay-sous-Bois, France). The hydrochloric acid standardized solution was purchased from Alfa Aesar (Kandel, Germany).

Phosphate buffered saline tablets were purchased from VWR International, LLC (Solon, OH, USA). Ethylenediaminetetraacetic acid disodium salt dehydrate was purchased from Sigma Chemical Co. (St. Louis, MO, USA); L(+)-ascorbic acid from Carl Roth GmbH+Co. KG (Karlsruhe, Germany), trichloroacetic acid glacial from VWR International, LLC (Leuven, Belgium), 2-thiobarbituric acid from Thermo Fisher (Kandel) GmbH (Karlsruhe, Germany), and 1,1,3,3-tetramethoxypropane from Tokyo Chemical Industry Co., Ltd. (Tokyo, Japan).

Strainer bags (BA6040/STR, 105 mm × 155 mm) were purchased from Seward Ltd. (Worthing, United Kingdom). Sodium chloride and plate count agar (granulated) were purchased from VWR Chemicals BDH Prolabo (Leuven, Belgium), Lactobacillus MRS agar (MRS agar) from HiMedia Laboratories Pvt. Ltd. (Mumbai, India), and iron agar (Lyngby) from Laboratorios Conda S.A. (Madrid, Spain).

### 2.2. Preparation and Treatment of Fish Samples

After being immediately slaughtered by percussive stunning, fishes were gutted and washed in potable water. The gutted fishes were then transferred to the laboratory within 20 min of slaughtering, and packed in insulated boxes containing ice. Here, fishes were further headed, filleted, and skinned by hand.

The prepared fillets were cut and then minced using a kitchen meat grinder (Philips Food processor HR7764/13, Amsterdam, Netherlands) equipped with a plate disc knife (4mm diameter holes). The minced fish meat was weighed into 15 ± 0.01 g portions; these were shaped into patties of approximately 43 mm diameter and 6 mm thickness using a customized burger mold.

The fish patties were divided into three batches (90 patties per batch); samples from the first batch were covered (top and bottom) with whey protein isolate-based films incorporated with 2.5% tarragon essential oil (WPIf+2.5%TEO), from the second batch with whey protein isolate-based films (WPIf), and from the third batch were left uncovered (Control). Next, all fish samples were aerobically packed into 100 × 150 mm sterile polyethylene ziplock bags and stored at 4 °C for 15 days. Physicochemical, microbiological and sensory analyses were performed at 3-day intervals to measure the quality of brook trout.

### 2.3. Preparation of Films

Two types of films (with and without tarragon essential oil) were prepared from heat-denatured whey protein isolate solutions. Film-forming solutions were obtained by dissolving 5% (*w*/*w*) WPI in distilled water, according to the protocol described by Socaciu et al. (2020) [6]. Glycerol was added to filmogenic solutions at a concentration of 5% (*w*/*w*).

Solutions were subsequently heated for 30 min at 90 ± 2 °C while being continuously stirred using a magnetic stirrer with heating (MSH-300, Biosan Ltd., Riga, Latvia). Heated solutions were then cooled at room temperature for 1.5 h and filtered to remove any air incorporated during stirring. For the preparation of active packaging films, the essential oil of tarragon was added to their solution to reach a final concentration of 2.5% (*w*/*w*).

Next, both solutions (with and without tarragon essential oil) were homogenized at 23,000 rpm for 2.5 min using a laboratory dispenser (T 18 digital Ultra-Turrax, IKA-Werke GmbH & Co. KG, Staufen, Germany). The final film-forming solutions were poured (4.8 g) into disposable weighing dishes (43 × 13 mm) and then dried in an oven (Digitheat, J.P. Selecta S.A., Barcelona, Spain) at 37 °C for 42 h. Once formed, the films were peeled off and conditioned for 72 h at 20 ± 2 °C and 50 ± 2% relative humidity in a climatic test cabinet (TK 252; Nüve Sanayi Malzemeleri İmalat ve Ticaret A.Ş., Akyurt/Ankara, Turkey), then used to cover the brook trout samples.

### 2.4. GC-MS Analysis of Essential Oil

The GC-MS analysis (gas chromatography coupled with mass spectrometry) of the volatile profile of tarragon essential oil was carried out as described in our previous paper, with some modification [23]. In this regard, the tarragon essential oil sample was diluted in n-hexane before the injection into the GC-MS injector.

A Zebron ZB-5 ms capillary column (30 m × 0.25 mm i.d. × 0.25 μm film thickness; Phenomenex, Torrance, CA, USA) was used for the chromatographic separation of volatile constituents. The column oven temperature program was set as follows, from 50 °C (kept at this temperature for 2 min) to 160 °C at 4 °C/min, then raised to 250 °C at 10 °C/min (kept at this temperature for 10 min). The temperature of the ion source, injector, and interface was set at 250 °C. Helium was used as the carrier gas at a flow rate of 1 mL/min. The split ratio was 1:200. The ion trap mass spectrometer was operated in EI-MS mode; the acquisition mode was set in the range of 40–500 *m*/*z*.

The volatile compounds were tentatively identified by comparing their recorded mass spectra with those of standard compounds (*β*-myrcene, limonene, 1,8-cineol, *α*-pinene, and caryophyllene) and with those from NIST27 and NIST147 spectra libraries (considering a minimum similarity of 95%) as well as by retention indices obtained from www.pherobase.com (accessed on 29 April 2020) [24] or www.flavornet.org (accessed on 29 April 2020) (for columns with a similar stationary phase to ZB-5 ms) [25]. The results were expressed as the relative percentage of each compound from the total ion chromatograms (TIC) area (100%).

### 2.5. Physicochemical Analysis of Fish

#### 2.5.1. Determination of Proximate Composition

The fat content, protein content, and moisture of the fish sample were measured using a near-infrared meat analyzer (FoodScan, FOSS, Hillerød, Denmark). Two replicates were run for each fish sample. The results were displayed as g/100 g fish sample.

The ash content was determined by incineration of the fish sample in a muffle furnace (L3/11/B170, Nabertherm GmbH, Bremen, Germany), according to the method described by Nagy et al. (2017) [26]. A 3.0 g portion of the fish sample was weighed in a porcelain melting pot and kept at 600 °C for 6 h in the muffle furnace. The ash content was calculated with the following Equation (1):

(1) $$\mathrm{Ash}\;\left(g/100\;g\;\mathrm{fish}\;\mathrm{sample}\right)=\frac{w_{a}}{w_{fs}}\times 100$$

where $w_{a}$ is the weight of the ash (g) and $w_{fs}$ is the weight of the fish sample (g). Two replicates were run for each fish sample.

#### 2.5.2. Determination of pH and Electrical Conductivity (EC)

The pH and EC of the fish sample were determined according to the method described in the ISO 2917:1999 standard [27]. A 10.0 g portion of the fish sample was homogenized in 100 mL distilled water for 30 sec using a glass rod. The mixture was left undisturbed at room temperature for 30 min and then filtered. The pH and EC of the extract were measured using a digital multi-parameter meter (InoLab^®^ Multi 9310 IDS, WTW, Weilheim, Germany). Two replicates were run for each fish sample. The result for EC was expressed in µS/cm.

#### 2.5.3. Determination of Total Volatile Basic Nitrogen (TVB-N)

The TVB-N concentration of the fish sample was determined by the reference procedure described in the European Commission’s Decision 95/149/EC [28]. A 10.0 g portion of the fish sample was homogenized in 90 mL of 6% (*w*/*v*) perchloric acid solution at 20,000 rpm for 2 min using a laboratory dispenser (T 18 digital Ultra-Turrax, IKA-Werke GmbH & Co. KG, Staufen, Germany). The mixture was then filtered and the obtained extract kept in the refrigerator until use.

A 50 mL aliquot of the extract was mixed with a few drops of silicone antifoaming agent and 6.5 mL of 20% (*w*/*v*) sodium hydroxide solution. The mixture was subjected to steam distillation using a Kjeldahl distillation unit (UDK 140, VELP Scientifica, Milan, Italy) till 100 mL of distillate had been produced. The distillate was collected in a flask containing 100 mL of 3% (*w*/*v*) boric acid solution and 3-5 drops of Tashiro mixed indicator (0.2 g methyl red and 0.1 g methylene blue dissolved in 100 mL 95% ethanol). Next, the receiver solution was titrated with a 0.01 N hydrochloric acid standardized solution until the endpoint (pH 5.0 ± 0.1) was reached. A blank sample was prepared with 50 mL of 6% (*w*/*v*) perchloric acid solution instead of extract and treated identically to the fish sample. The TVB-N concentration was calculated with the following Equation (2):

(2) $$\mathrm{TVB}-N\;\left(\mathrm{mg}\;N/100\;g\;\mathrm{fish}\;\mathrm{sample}\right)=\frac{(V_{1}-V_{0})\times 0.14\times 2\times 100}{w_{fs}}$$

where $V_{1}$ is the volume of 0.01 N hydrochloric acid solution consumed for the fish sample at endpoint (mL), $V_{0}$ is the volume of 0.01 N hydrochloric acid solution consumed for the blank at endpoint (mL), and $w_{fs}$ is the weight of the fish sample (g). Three replicates were run for each fish sample.

#### 2.5.4. Determination of Thiobarbituric Acid Reactive Substances (TBARS)

The TBARS concentration of the fish sample was determined according to the modified method of Semeniuc et al. (2016a, 2016b) [29,30].

A 2.0 g grams portion of the fish sample was homogenized in 4 mL of PBS [phosphate buffered saline (1X solution, pH 7.4) containing 0.1% (*w*/*v*) ethylenediaminetetraacetic acid disodium salt and 0.1% (*w*/*v*) ascorbic acid] at 21,500 rpm for 30 sec using a laboratory dispenser (T 18 digital Ultra-Turrax, IKA-Werke GmbH & Co. KG, Staufen, Germany); 2 mL of TCA [30% (*w*/*v*) trichloroacetic acid solution] was then added and again homogenized at 17,500 rpm for 30 sec. The mixture was transferred into a 10-mL volumetric flask, brought to volume with PBS and vortexed (Vortex V-1 Plus, Biosan Ltd., Riga, Latvia). The precipitate formed was removed by filtration, using a Whatman no. 1 filter paper, and the extract was collected.

A 5 mL aliquot of the extract was mixed with 5 mL of TBA [0.02 M 2-thiobarbituric acid solution] into a 10-mL test tube with screw cap. The test tube was immersed in a water bath at 90 °C and kept it for 20 min. Subsequently, the test tube was placed into a refrigerator (~4 °C) for 30 min to cool. A blank sample was prepared with 4 mL of PBS and 1 mL of TCA instead of extract and treated identically to the fish sample. The absorbance of each fish sample was read against the blank at 530 nm using a double beam UV-Vis spectrophotometer (UV-1900i, Shimadzu Scientific Instruments, Inc., Columbia, MD, USA). Three replicates were run for each fish sample.

Seven standard solutions of 1,1,3,3-tetra methoxy propane (TMP), a precursor of malondialdehyde, were prepared and subjected to the TBARS assay to construct a calibration curve. The calibration curve was linear over the range of 1.88 to 112.80 nmol MDA/mL. The TBARS concentration was calculated with the following Equation (3):

(3) $$\mathrm{TBARS}\;\left(\mathrm{mg}\;\mathrm{MDA}/\mathrm{kg}\;\mathrm{fish}\;\mathrm{sample}\right)=\frac{\left(Abs.\;-b\right)}{m}\times 10^{-10}\times \frac{10}{w_{fs}}\times 72.06\times 10^{6}$$

where Abs. is the absorbance value at 530 nm, b is the y-intercept of the linear equation, m is the slope of the regression line, 10 is the volume of the mixture before filtration (mL), $w_{fs}$ is the weight of the fish sample (g), and 72.06 is the molecular weight of malondialdehyde (g/mol).

#### 2.5.5. Measurement of Color

The color of the fish sample was measured using an NH300 portable colorimeter (3NH, Shenzhen, China) based on the CIE L*a*b* color system. The L*-value represents lightness and ranges from zero (the darkest black) to 100 (the brightest white). The a*-value represents “redness” or “greenness” and ranges from +60 for absolute red to −60 for absolute green, while b*-value represents “yellowness” or “blueness” and ranges from +60 for absolute yellow to −60 for absolute blue. Measurements were performed using a D65 illuminant with an opening of 8 mm and a 10° standard observer. The colorimeter was subjected to automatic black and white calibration. Twelve readings were taken on each fish sample.

### 2.6. Microbiological Analysis of Fish

An amount of 5.0 g fish sample was aseptically weighed into a sterile stomacher bag and homogenized in 45 mL of 0.85% (*w*/*v*) sodium chloride solution for 1 min using a bag mixer (MiniMix^®^ 100 P CC^®^, Interscience, Saint Nom, France). Seven ten-fold serial dilutions (10^−2^, 10^−3^, 10^−4^, 10^−5^, 10^−6^, 10^−7^, and 10^−8^) were prepared from this stock solution (10^−1^).

The total viable count (TVC) and psychrotrophic count (PTC) were determined according to the method described in the ISO 4833-1:2013 standard using plate count agar [31]. Enumeration of TVC and TPC was performed after incubation of inoculated plates at 30 °C for 72 h [31], respectively at 7 °C for 10 days [32].

Lactic acid bacteria (LAB) were determined according to the method described in the ISO 7889|IDF 117:2003 standard using MRS agar [33]. Enumeration of colonies was performed after anaerobic incubation of inoculated plates at 37 °C for 72 h.

Hydrogen sulfide (H_2_S)-producing bacteria (including *Shewanella putrefaciens*) were determined according to the method described by Yu et al. (2017) using iron agar in double-layer [34]. Enumeration of colonies (*Pseudomonas fluorescens* as white colonies and *Shewanella putrefaciens* as black colonies) was performed after incubation of inoculated plates at 30 °C for 4 days.

All counts were expressed as log_10_ CFU/g and performed in duplicate.

### 2.7. Sensory Evaluation of Fish

Sensory evaluation was carried out as described by Bahram et al. (2016), with minor modification [16]. The fish samples were evaluated by 5 trained panelists from the laboratory staff (four women and one man, aged 25–41 years). The fish samples were taken out of the bags and films removed from the covered ones. These were then individually placed on small white ceramic plates, coded with 3-digit random numbers, and presented to panelists. They were asked to assess the patties in the following order: (1) the fish patty from control batch, (2) the fish patty from WPIf batch, and (3) the fish patty from WPIf+2.5%TEO batch. The judges were not informed about the batch and storage time of fish patties. The panelists were asked not to eat, drink, or smoke for at least 1 h before the evaluation session; they evaluated the sensory attributes of fish patties at 20 °C (air -conditioning) under white light, in individual cabins.

The sensory evaluation was based on a five-point scale to determine: color discoloration (5, no discoloration; 4, slight discoloration; 3, moderate discoloration; 2, strong discoloration; 1, extreme discoloration), odor (5, extremely desirable; 4, slightly desirable; 3, neither desirable nor unacceptable/off-odor; 2, slightly unacceptable/off-odor; 1, extremely unacceptable/off-odor), and overall acceptability (5, extremely desirable; 4, slightly desirable; 3, neither desirable nor unacceptable; 2, slightly unacceptable; 1, extremely unacceptable). Shelf-life criteria assumed that rejection would occur when the sensory attributes declined below 4.0.

### 2.8. Statistical Analysis

Data analysis was carried out using Minitab statistical software (version 16.1.0, LEAD Technologies, Inc., Charlotte, NC, USA). The differences in results among different groups were determined using one-way ANOVA. Post-hoc pairwise comparisons were performed with Tukey’s test at a 95% confidence level (*p* < 0.05). Correlations among data were calculated using Pearson’s correlation coefficient.

## 3. Results and Discussion

### 3.1. Volatile Composition of Essential Oil

The volatile compounds detected in tarragon essential oil by GC-MS analysis are listed in Table 1. Nine compounds, representing 100% of the total detected constituents, were identified in the essential oil of tarragon and grouped based on their chemical structure into four classes (monoterpene hydrocarbons, oxygenated monoterpenes, phenylpropanoids, and sesquiterpene hydrocarbons). The most abundant constituents were phenylpropanoids (81.84%), followed by monoterpene hydrocarbons (17.47%), the oxygenated monoterpenes (0.42%), and sesquiterpene hydrocarbons (0.27%). The major components identified in tarragon essential oil were estragole (81.84%), cis-*β*-ocimene (6.51%), trans-*β*-ocimene (6.19%), and D-limonene (4.05%).

### 3.2. Physicochemical Properties of Fish

#### 3.2.1. Proximate Composition

The results of the proximate analysis of brook trout showed a mean value of 19.8 ± 0.078 g/100 g fish sample for protein content, 8.0 ± 0.255 g/100 g fish sample for fat content, 1.4 ± 0.014 g/100 g fish sample for ash content, and 70.2 ± 0.290 g/100 g fish sample for moisture content. The values reported in other studies [35,36] showed some differences, especially in the lipid and protein contents. Variations in the chemical composition of fish muscle among individuals in the same species are related to the state of nutrition, the reproductive cycle of the animal, fish size, as well as other environmental conditions [37].

#### 3.2.2. pH and Electrical Conductivity (EC)

The pH value is an indicator of the freshness of meat, which is fundamental to fish quality [38]. In the flesh of live fish, pH is close to 7.0. After their death, pH can vary from 6.0 to 7.1, depending on the season, species, and other factors [16].

**Figure 1:**
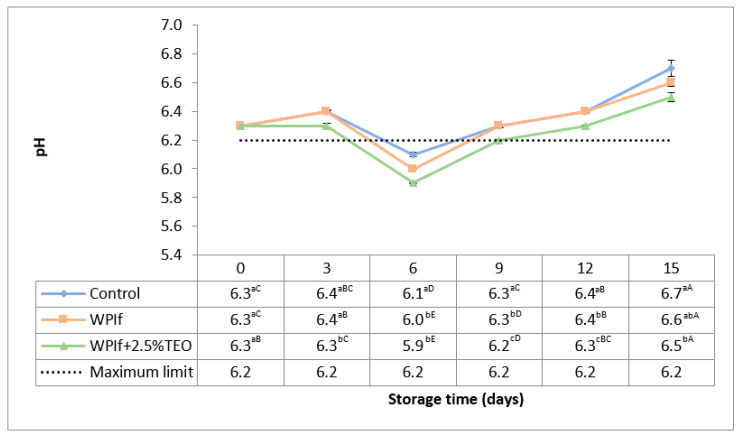
Changes in pH of brook trout samples during refrigerated storage. Control-uncovered fish samples; WPIf-fish samples covered with WPI-based films; WPIf+2.5%TEO-fish samples covered with whey protein isolate-based films incorporated with 2.5% tarragon essential oil. Values are expressed as mean ± standard deviation of two replicates. Means that do not share a letter (lowercase letters on column and uppercase letters on row) are significantly different.

The values of pH in brook trout samples stored at 4 °C for 15 days are shown in Figure 1. Changes in pH showed the same trend during storage in all treatments. The initial value of pH in the fish sample was 6.3, comparable with the value (of 6.5) found by Shen et al. (2015) [39] in rainbow trout fillets but lower than values (of 7.0 and 7.2, respectively) reported by Nistor et al. (2014) [40] and Linhartová et al. (2018) [36] in brook trout fillets. The pH stayed relatively stable up to the third day of storage (6.4 in the control sample, 6.4 in WPIf sample, and 6.3 in WPIf+2.5%TEO sample), decreased from the third day to the sixth day of storage (to 6.1 in the control sample, 6.0 in WPIf sample, and 5.9 in the WPIf+2.5%TEO sample), then increased up to the 15th day of storage (to 6.7 in the control sample, 6.6 in WPIf sample, and 6.5 in WPIf+2.5%TEO sample). The initial decrease in pH could be attributed to the post-mortem breakdown of glycogen, ATP, creatine phosphate [39,41,42], and the dissolution of CO_2_ in the fish sample [43]; the later increase in pH was probably due to the production of volatile bases [39,41,44]. A similar decreasing-increasing pH trend during refrigerated storage was also reported by Shen et al. (2015) in rainbow trout fillets [39].

There were significant differences in pH values between batches from the 3rd day to the 15th day of storage; the lowest pH values were found in WPIf+2.5%TEO samples, followed by WPIf samples, and by control samples. These results show that films, particularly the active film, delayed enzymes activity keeping thus the freshness of fish samples.

The maximum permitted value for pH in fresh fish is 6.2, as set by Romanian standard STAS 5386-86 [45]. The value of pH exceeded the limit level in the 9th day of storage for control and WPIf samples, respectively, in the 12th day of storage for the WPIf+2.5%TEO sample.

EC is an index of the concentration of electrolytes in the muscle tissue of fish; it can impact body fluid balance, survival, and meat quality [17,39]. The autolytic spoilage that post-mortem occurs in fish, mainly caused by enzymes, progressively disrupts the muscle cell membranes [46]. As a consequence, the intracellular fluid leak into the intercellular space and, being an electrolyte solution, increases the EC of the tissue [47].

**Figure 2:**
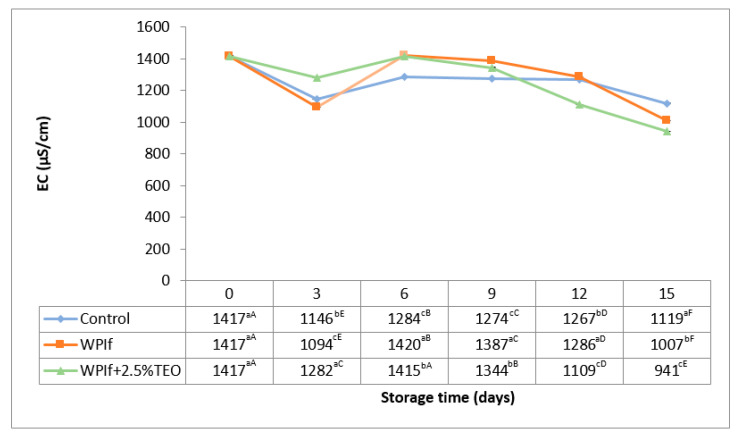
Changes in electrical conductivity (EC) of brook trout samples during refrigerated storage. Control-uncovered fish samples; WPIf-fish samples covered with WPI-based films; WPIf+2.5%TEO-fish samples covered with whey protein isolate-based films incorporated with 2.5% tarragon essential oil. Values are expressed as mean ± standard deviation of two replicates. Means that do not share a letter (lowercase letters on column and uppercase letters on row) are significantly different.

An opposite behavior to that of pH was noticed for electrical conductivity in fish samples during storage (see Figure 2); hence the strong negative correlation (r = −0.688; *p* < 0.05) found between pH and EC values. The initial value of EC in the fish sample was 1417 µS/cm, comparable with that reported by Shen et al. (2015) in rainbow trout fillets (1344 µS/cm) [39]. The EC value decreased in the 3rd day of storage (to 1146 µS/cm in the control sample, to 1094 µS/cm in WPIf sample, and to 1282 µS/cm in WPIf+2.5%TEO sample), then increased from the 3rd day to the 6th day of storage (to 1284 µS/cm in the control sample, to 1420 µS/cm in WPIf sample, and to 1415 µS/cm in WPIf+2.5%TEO sample), and decreased again up to the 15th day of storage (to 1119 µS/cm in the control sample, to 1007 µS/cm in WPIf sample, and to 941 µS/cm in WPIf+2.5%TEO sample). In the final stages of storage, slight drip losses were observed in fish samples. This could be the reason for the reduction of electrolyte concentration in fish samples. Contrary to our findings, other studies have reported an increase in EC of rainbow trout [39] and beluga sturgeon fillets [16] during refrigerated storage. The mobility ratios of positive and negative ions in the fish patties could be the reason for “in the mirror” behaviors of EC and pH during refrigerated storage, negative ions being smaller and more mobile than positive ions [48,49]. Variations of pH and EC up to the 6th day of refrigerated storage are due to pre-rigor, in-rigor, and post-rigor changes in brook trout samples.

#### 3.2.3. Total Volatile Basic Nitrogen (TVB-N)

The TVB-N quantifies the presence of nitrogenous compounds (ammonia, dimethyl amine, and trimethyl amine) in fish from the sea or from river, revealing the degree of freshness [50]. Its increase during storage is related to the activity of spoilage bacteria and endogenous enzymes [32]. The Commission of the European Union, through the Regulation (EC) No. 1022/2008, [51] has set limit values for TVB-N just in redfish, flatfish, Atlantic salmon, hake, and gadoids; values ≤25 mg N/100 g flesh for *Sebastes* spp., *Helicolenus dactylopterus*, and *Sebastichthys capensis*, ≤30 mg N/100 g flesh for species belonging to the Pleuronectidae family (with the exception of halibut: *Hippoglossus* spp.), and ≤35 mg N/100 g flesh for *Salmo salar*, species belonging to the Merlucciidae family, and species belonging to the Gadidae family. However, the TVB-N value as an indicator of fish freshness has been recently disputed as it was reported below the maximum limit, even when the fish has been rejected by sensory evaluation [52].

**Figure 3:**
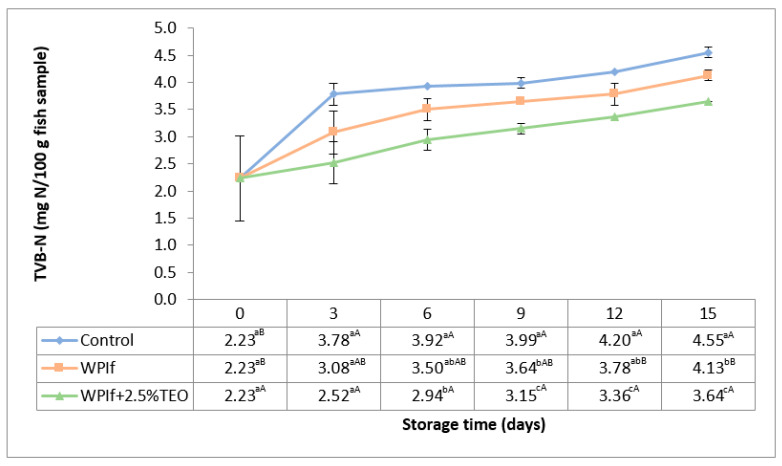
Changes in total volatile basic nitrogen (TVB-N) of brook trout samples during refrigerated storage. Control-uncovered fish samples; WPIf-fish samples covered with WPI-based films; WPIf+2.5%TEO-fish samples covered with whey protein isolate-based films incorporated with 2.5% tarragon essential oil. Values are expressed as mean ± standard deviation of three replicates. Means that do not share a letter (lowercase letters on column and uppercase letters on row) are significantly different.

The TVB-N values of brook trout samples during refrigerated storage are presented in Figure 3. Initially, the TVB-N value was 2.23 mg N/100 g fish sample; a comparable value (3.59 mg N/100 mg fish sample) was found by Feng et al. (2016) in golden pomfret fillets [17] and much higher values by Kazemi and Rezaei (2015) in rainbow trout slices (10.37 mg N/100 g fish sample) [15], by Bahram et al. (2016) in beluga sturgeon fillets (17.97 mg N/100 mg fish sample) [16], and by Shokri and Ehsani (2017) in pike-perch fillets (10.99 mg N/100 g fish sample) [18]. To our knowledge, no prior studies have investigated the level of TVB-N in brook trout during storage.

The TVB-N value significantly increased with storage time from 2.23 to 4.55 mg N/100 g fish sample in the control batch, from 2.23 to 4.13 mg N/100 g fish sample in WPIf batch, and from 2.23 to 3.64 mg N/100 g fish sample in WPIf+2.5%TEO batch. Beginning with the sixth day of storage, there were significant differences in TVB-N values between batches; the lowest TVB-N values were found in WPIf+2.5%TEO samples, followed by WPIf samples, and by control samples. These results corroborate the findings from pH measurements and show that fish samples covered with films were more protected toward protein degradation, particularly those covered with active films. Since no limit of acceptability for TVB-N in brook trout has been established by the European Commission [51] or proposed by other researchers, this parameter was not used in establishing the shelf-life of brook trout from the current study.

#### 3.2.4. Thiobarbituric Acid Reactive Substances (TBARS)

The TBARS value is commonly used as an indicator of lipid oxidation, particularly in meat and fish products [53]; thiobarbituric acid reactive substances are formed in the second stage of auto-oxidation when peroxides are oxidized to aldehydes and ketones [54]. Some researchers [18,32,55] have proposed quality criteria for fish: <3 mg MDA/kg for perfect quality material, 3 ≤ mg MDA/kg < 5 for good quality material, and 5 ≤ mg MDA/kg < 8 for suitable for human consumption material. Nevertheless, these thresholds have not yet received regulatory approval.

**Figure 4:**
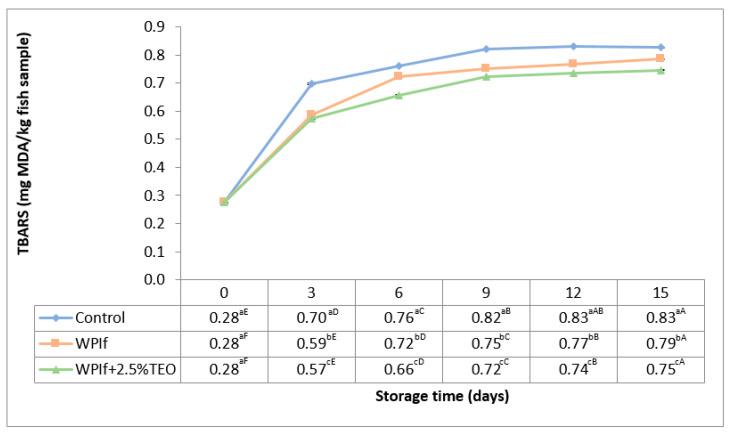
Changes in thiobarbituric acid reactive substances (TBARS) of brook trout samples during refrigerated storage. Control-uncovered fish samples; WPIf-fish samples covered with WPI-based films; WPIf+2.5%TEO-fish samples covered with whey protein isolate-based films incorporated with 2.5% tarragon essential oil. Values are expressed as mean ± standard deviation of three replicates. Means that do not share a letter (lowercase letters on column and uppercase letters on row) are significantly different.

The TBARS values of fish samples during refrigerated storage are shown in Figure 4. The initial value of TBARS in the fish sample was 0.28 mg MDA/kg (perfect quality material); comparable initial levels were reported by Ojagh et al. (2010) in rainbow trout fillets (0.09 mg MDA/kg) [55], by Li et al. (2013) in red drum fillets (0.28 mg MDA/kg) [37], by Jouki et al. (2014) in rainbow trout fillets (0.12 mg MDA/kg) [32], by Ramezani et al. (2015) in silver carp fillets (0.51 mg MDA/kg) [54], by Bahram et al. (2016) in beluga sturgeon fillets (0.02 mg MDA/kg) [16], by Shokri and Ehsani (2017) in pike-perch fillets (0.61 mg MDA/kg) [18], and by Yu et al. (2017) in grass carp fillets (0.20 mg MDA/kg) [34].

The TBARS value significantly increased with storage time from 0.28 to 0.83 mg MDA/kg fish sample in the control batch, from 0.28 to 0.79 mg MDA/kg fish sample in WPIf batch, and from 0.28 to 0.75 mg MDA/kg fish sample in WPIf+2.5%TEO batch. From the 3rd day of storage, there were significant differences in TBARS values between batches; the lowest TBARS values were found in WPIf+2.5%TEO samples, followed by WPIf samples, and by control samples. These results show that fish samples covered with films were less susceptible to lipid oxidation, especially those covered with active films. The TBARS values for all fish samples were below the upper proposed limits throughout the 15-day storage period.

#### 3.2.5. Color

**Figure 5:**
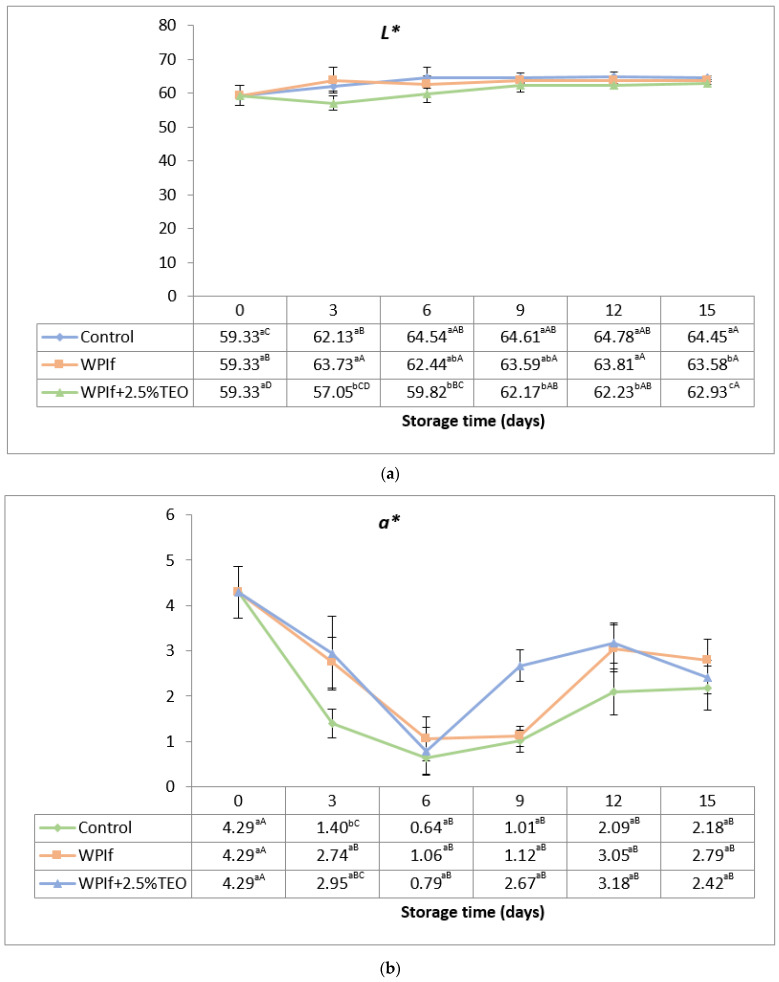

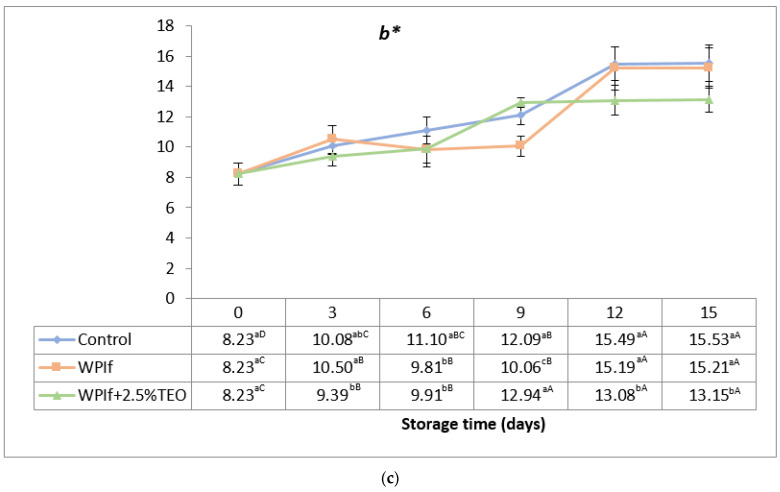
Changes in color attributes of brook trout samples during refrigerated storage. (**a**) L*; (**b**) a*; (**c**) b*. Control-uncovered fish samples; WPIf-fish samples covered with WPI-based films; WPIf+2.5%TEO-fish samples covered with whey protein isolate-based films incorporated with 2.5% tarragon essential oil. Values are expressed as mean ± standard deviation of twelve replicates. Means that do not share a letter (lowercase letters on column and uppercase letters on row) are significantly different.

Changes in color attributes (L*, a*, and b*) of brook trout samples during refrigerated storage are shown in Figure 5a–c. The initial values of L*, a*, and b* in the fish sample were 59.33, 4.29, and 8.23, respectively. Shen et al. (2005) have reported initial values of 62.77 for L*, 7.48 for a*, and 20.92 for b* in the brook trout fillet [39], therefore higher.

The L*-value (brightness) significantly increased with storage time from 59.33 to 64.45 in the control batch, from 59.33 to 63.58 in WPIf batch, and from 59.33 to 62.93 in WPIf+2.5%TEO batch (see Figure 5a). The values of L* in WPIf+2.5%TEO samples were significantly lower than those in WPIf and control samples, beginning with the third day of storage; there were no significant differences in L*-values between control and WPIf samples up to the 12th day of storage.

Variations of a*-values (redness) showed the same trend during storage for all batches (see Figure 5b). The initial value of a* in the fish sample was 4.29. It decreased up to the sixth day of storage (to 0.64 in the control sample, 1.06 in WPIf sample, and 0.79 in WPIf+2.5%TEO sample), then increased up to the 15th day of storage (to 2.18 in the control sample, 2.79 in WPIf sample, and 2.42 in WPIf+2.5%TEO sample). The reasons for these oscillations are not yet entirely understood. The initial decrease could be caused by the oxidation of red pigments, such as myoglobin and hemoglobin. A possible explanation for the later increased might be the pigments concentrating, as a result of dehydration. The values of a* in control samples were lower than those in WPIf and WPIf+2.5%TEO samples, but significantly only in the third day of storage; there were no significant differences in a*-values between WPIf and WPIf+2.5%TEO samples.

The b*-value (yellowness) significantly increased with storage time, probably due to the oxidation of lipids; from an initial value of 8.23 to 15.53 in the control batch, 15.21 in WPIf batch, and 13.15 in WPIf+2.5%TEO batch (see Figure 5c). There were significant differences in b*-values between batches from the third day to the ninth day of storage; the values of b* were generally lower in WPIf+2.5%TEO samples than those in WPIf and control samples.

The strong positive correlation (r = 0.704; *p* < 0.05) found between L*- and b*-values indicates that the brightness of fish samples increased with their yellowing. Given that L*-values increased and a*-values oscillated during storage, a moderate negative correlation (r = −0.511; *p* < 0.05) was found between L*- and a*-values. No significant correlation (r = −0.164; *p* ≥ 0.05) was found between a*- and b*-values; thus, no relationship between redness and yellowness of the fish samples.

All these results indicate a discoloration of fish samples (increase in brightness) with storage time caused by oxidation of red pigments (decrease in redness) and lipids (increase in yellowness); the least affected were samples covered with whey protein isolate-based films incorporated with 2.5% tarragon essential oil.

### 3.3. Microbiological Properties of Fish

#### 3.3.1. Total Viable Count (TVC)

**Figure 6:**
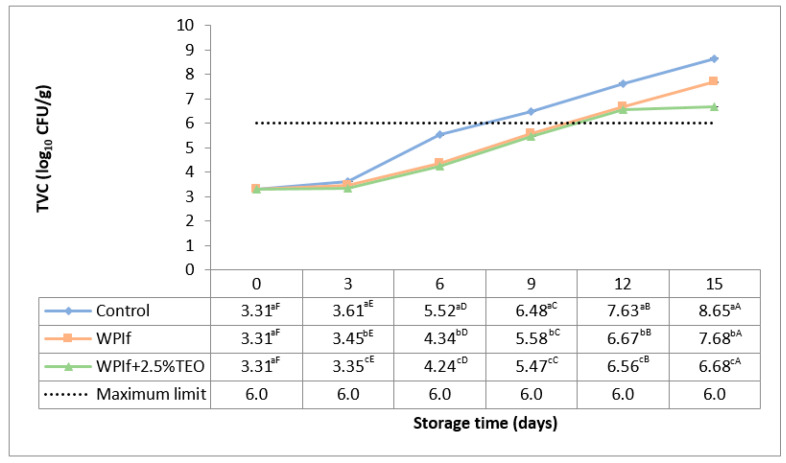
Changes in total viable count (TVC) of brook trout samples during refrigerated storage. Control-uncovered fish samples; WPIf-fish samples covered with WPI-based films; WPIf+2.5%TEO-fish samples covered with whey protein isolate-based films incorporated with 2.5% tarragon essential oil. Values are expressed as mean ± standard deviation of two replicates. Means that do not share a letter (lowercase letters on column and uppercase letters on row) are significantly different.

The total viable count (TVC) estimates the total number of aerobic mesophilic organisms (such as bacteria, yeasts, and molds) in the fish sample [56]. Changes in TVC of brook trout samples stored at 4 °C for 15 days are shown in Figure 6. The initial TVC in the fish sample was 3.31 log_10_ CFU/g. Comparable initial counts were reported by Ojagh et al. (2010) [55], Jouki et al. (2014) [32], respectively Volpe et al. (2015) [52] in rainbow trout fillets (3.86 log_10_ CFU/g, 3.58 log_10_ CFU/g, and 4.00 log_10_ CFU/g), by Li et al. (2013) in red drum fillets (3.92 log_10_ CFU/g) [37], by Kazemi and Rezaei (2015) in rainbow trout slices (2.50 log_10_ CFU/g) [15], by Bahram et al. (2016) in beluga sturgeon fillets (4.04 log_10_ CFU/g) [16], and by Yu et al. (2017) in grass carp fillets (4.20 log_10_ CFU/g) [34]. The TVC stayed relatively stable up to the third day of storage (3.61 log_10_ CFU/g in the control sample, 3.45 log_10_ CFU/g in WPIf sample, and 3.35 log_10_ CFU/g in WPIf+2.5%TEO sample), then significantly increased up to the 15th day of storage (to 8.65 log10 CFU/g in the control sample, 7.68 log10 CFU/g in WPIf sample, and 6.68 log_10_ CFU/g in WPIf+2.5%TEO sample). Generally, TVC increased with increasing pH in fish samples with decreasing EC; from here there was a moderate positive correlation with the pH (r = 0.518; *p* < 0.05) and moderate negative correlation (r = −0.517; *p* < 0.05) with the EC.

There were significant differences in total viable counts between batches from the third day to the 15th day of storage with the lowest values in WPIf+2.5%TEO samples, followed by WPIf samples, and by control samples. These findings show that films, particularly the one incorporated with tarragon essential oil, inhibited microbial growth.

According to the Food Safety Authority of Ireland [57], the level of TVC in refrigerated fish should be less than 10^6^ CFU/g (6.0 log_10_ CFU/g). The TVC exceeded the limit level in the 9th day of storage for the control sample, respectively in the 12th day of storage for WPIf and WPIf+2.5%TEO samples.

#### 3.3.2. Psychrotrophic Count (PTC)

**Figure 7:**
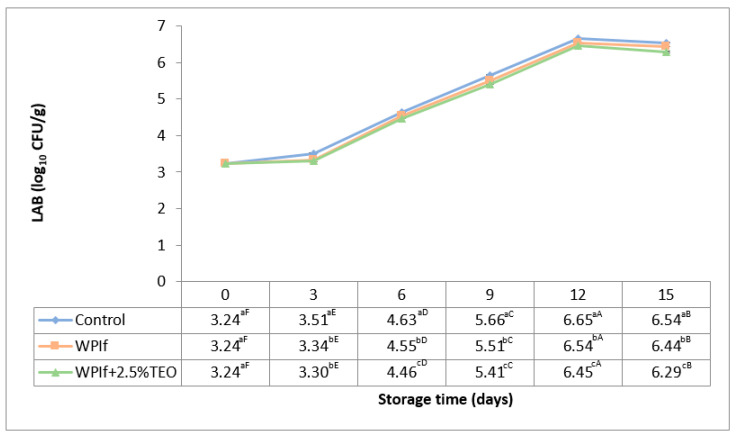
Changes in psychrotrophic count (PTC) of brook trout samples during refrigerated storage. Control-uncovered fish samples; WPIf-fish samples covered with WPI-based films; WPIf+2.5%TEO-fish samples covered with whey protein isolate-based films incorporated with 2.5% tarragon essential oil. Values are expressed as mean ± standard deviation of two replicates. Means that do not share a letter (lowercase letters on column and uppercase letters on row) are significantly different.

The spoilage of aerobically stored fish is mainly due to the Gram-negative psychrotrophic non-fermenting rods [58]. These bacteria are capable of growth at 0 °C but with optimum around 25 °C [41]. Therefore, psychrotrophic bacteria were counted in brook trout samples during 15 days of storage at 4 °C (see Figure 7).

The growth pattern of PTC showed the same behavior in all treatments. The initial value of PTC in the fish sample was 3.24 log_10_ CFU/g. Other researchers have found initial levels of 2.88 log_10_ CFU/g and 3.10 log_10_ CFU/g in rainbow trout fillets [32,55], of 2.50 log_10_ CFU/g in rainbow trout slices [15], of 3.56 log_10_ CFU/g in silver carp fillets [54], of 3.89 log_10_ CFU/g in beluga sturgeon fillets [16], of 3.18 log_10_ CFU/g in pike-perch fillets [18], respectively of 3.50 log_10_ CFU/g in grass carp fillets [34]. The TPC significantly increased with storage time from 3.24 to 8.57 log_10_ CFU/g in the control batch, from 3.24 to 7.64 log_10_ CFU/g in WPIf batch, and from 3.24 to 7.49 log_10_ CFU/g in WPIf+2.5%TEO batch. From the third day of storage, significant differences were found between total viable counts of fish samples; the values of WPIf+2.5%TEO samples were lower than those of WPIf and control samples.

#### 3.3.3. Lactic Acid Bacteria (LAB)

**Figure 8:**
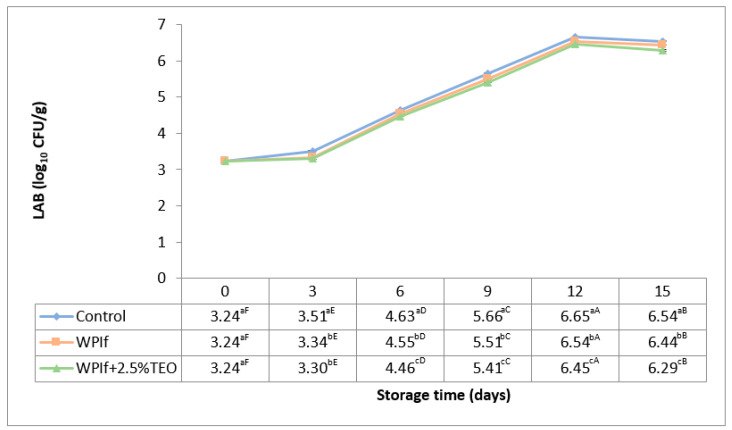
Changes in lactic acid bacteria (LAB) of brook trout samples during refrigerated storage. Control-uncovered fish samples; WPIf-fish samples covered with WPI-based films; WPIf+2.5%TEO-fish samples covered with whey protein isolate-based films incorporated with 2.5% tarragon essential oil. Values are expressed as mean ± standard deviation of two replicates. Means that do not share a letter (lowercase letters on column and uppercase letters on row) are significantly different.

Lactic acid bacteria are also associated with the spoilage of fish during refrigerated storage [58]. Initial populations of 2.30 log_10_ CFU/g, respectively 2.00 log_10_ CFU/g were found in rainbow trout fillets by Jouki et al. (2014) and by Volpe et al. (2015) [32,52]. In rainbow trout slices, Kazemi and Rezaei (2015) have reported an initial count of 3.00 log_10_ CFU/g LAB [15]. Changes in LAB of brook trout samples during storage at 4 °C for 15 days are shown in Figure 8. These have shown the same trend during storage, to all treatments. The initial count of LAB in the fish sample was 3.24 log_10_ CFU/g. It significantly increased up to the 12th day of storage (to 6.65 log_10_ CFU/g in the control sample, 6.54 log_10_ CFU/g in WPIf sample, and 6.45 log_10_ CFU/g in WPIf+2.5%TEO sample), then significantly decreased (to 6.54 log_10_ CFU/g in the control sample, 6.44 log_10_ CFU/g in WPIf sample, and 6.29 log_10_ CFU/g in WPIf+2.5%TEO sample). The later decrease is probably due to the competition of LAB with other microorganisms from the matrix for the remained nutrients [59]. From the 3rd day of storage, there were significant differences between batches regarding LAB counts; the lowest values were found in WPIf+2.5%TEO samples, followed by WPIf samples, and by control samples.

#### 3.3.4. Hydrogen Sulfide (H_2_S)-Producing Bacteria

**Figure 9:**
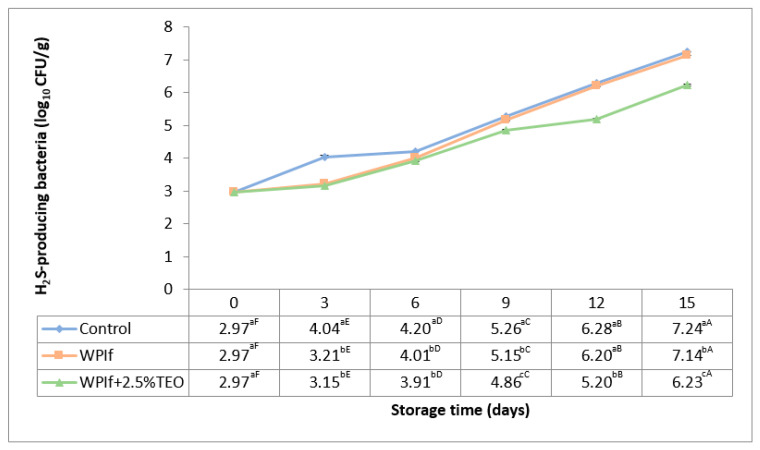
Changes in hydrogen sulfide (H_2_S)-producing bacteria of brook trout samples during refrigerated storage. Control-uncovered fish samples; WPIf-fish samples covered with WPI-based films; WPIf+2.5%TEO-fish samples covered with whey protein isolate-based films incorporated with 2.5% tarragon essential oil. Values are expressed as mean ± standard deviation of two replicates. Means that do not share a letter (lowercase letters on column and uppercase letters on row) are significantly different.

Some spoilage microorganisms in fish, including *Shewanella putrefaciens*, release hydrogen sulfide (H_2_S) upon decomposition of sulfur-containing amino acids [60]. These are so-called H_2_S-producing bacteria. Some researchers have reported initial counts of H_2_S-producing bacteria by 2.20 log_10_ CFU/g and 2.00 log_10_ CFU/g in rainbow trout fillets [32,52], and by 3.20 log_10_ CFU/g in grass carp fillets [34]. Changes in hydrogen sulfide (H_2_S)-producing bacteria of brook trout samples during refrigerated storage are shown in Figure 9. All batches revealed the same behavior during storage. The initial count of H_2_S-producing bacteria in the fish sample was 2.97 log_10_ CFU/g. It significantly increased with storage time, up to 7.24 log_10_ CFU/g in the control batch, 7.14 log_10_ CFU/g in WPIf batch, and 6.23 log_10_ CFU/g in WPIf+2.5%TEO batch. Starting with the third day of storage, significant differences were observed between counts of H_2_S-producing bacteria in fish samples; the values of WPIf+2.5%TEO samples were lower than those of WPIf and control samples.

### 3.4. Sensory Properties of Fish

The color discoloration, odor, and overall acceptability are the sensory attributes chosen to evaluate the quality of fish patties during refrigerated storage as these are significantly changed with fish spoilage. The lower the sensory score of an attribute, the lower the quality of the fish sample. The results of sensory evaluation of fish samples are given in Table 2. The fish samples were considered to be acceptable for human consumption up to a score of 4 [16,54,55]. Variations in scores of sensory attributes (color discoloration, odor, and overall acceptability) showed the same behavior during storage to all treatments; no change up to the sixth day of storage (scores of 5.0 points), then a significant decrease up to the 15th day of storage. From the sixth to the 15th day of storage, the score for color discoloration significantly decreased from 5.0 points to 1.0 point in the control sample, 2.0 points in WPIf sample, and 2.6 points in WPIf+2.5%TEO sample. There were significant differences in scores of color discoloration between all treatments; the lowest scores for color discoloration were found in control samples, followed by WPIf samples, and by WPIf+2.5%TEO samples. Strong negative correlations were found between color discoloration scores and L*-values (r = −0.605; *p* < 0.05) and between color discoloration scores and b*-values (r = −0.880; *p* < 0.05). These results strengthen the findings of color measurements.

In terms of odor and overall acceptability, the scores of WPIf+2.5%TEO samples were significantly higher than those of control and WPIf samples; no significant differences were found between scores of the two later batches. Between the sixth and the 15th day of storage, the scores for odor and overall acceptability significantly decreased from 5.0 points to 1.0 point in the control sample, 2.0 points in WPIf sample, and 2.0 points in WPIf+2.5%TEO sample. These results indicate a depreciation of the odor and appearance of fish samples with storage time, to the same extent in samples covered with films and to a greater extent in uncovered samples. To all sensory attributes, the unacceptable score was given on the ninth day of storage for the control sample and in the 12th day of storage for WPIf and WPIf+2.5%TEO samples.

### 3.5. Shelf-Life of Fish

The shelf-lives resulted from physicochemical, microbiological, and sensory evaluations of brook trout samples are summarized in Table 3. Based on the pH value, the shelf-life of fish sample covered with the active film was extended by three days (from six to nine days), but that of the fish sample covered with the control film was not improved. Based on the total viable count and sensory scores, both the shelf-life of WPIf sample and WPIf+2.5%TEO sample was prolonged by three days. Taking into consideration all parameters, a shelf-life of six days was achieved for the uncovered fish sample, of six–nine days for the fish sample covered with control film, and of nine days for the fish sample covered with active film. These findings demonstrate the research hypothesis of our study.

## 4. Conclusions

The WPI-based film incorporated with 2.5% tarragon essential oil has proven to be effective in preserving the quality and, thus, in improving the shelf-life of brook trout sample during storage at 4 °C. This film has shown to possess good antioxidant and antimicrobial properties. The tarragon essential oil from its matrix has caused delays of chemical reactions and microorganisms growth in the fish sample, leading to retention of desirable sensory attributes for a longer period. Due to the low level of incorporation didn’t negatively affect the organoleptic properties of the fish sample. The cost of raw materials for the manufacturing of 100 cm^2^ WPIf+2.5%TEO reaches 1.4 €. In summary, this active packaging material has good industrial application potential.

## Figures and Tables

**Table 1:** Relative contents (%) of volatile constituents identified in tarragon essential oil.

Crt. No.	Compound	Chemical Class	Retention Time	Relative Content
1	*α*-Pinene	M.Hc.	7.949	0.54
2	*β*-Myrcene	M.Hc.	9.920	0.18
3	**D-Limonene**	M.Hc.	11.406	**4.05**
4	1,8-cineole	O.M.	11.534	0.10
5	**trans-*β*-Ocimene**	M.Hc	11.664	**6.19**
6	**cis-*β*-Ocimene**	M.Hc	12.065	**6.51**
7	*α*-Terpineol	O.M.	17.787	0.32
8	**Estragole**	Phe.P.	17.917	**81.84**
9	Caryophyllene	S.Hc.	25.869	0.27
-	TOTAL	-	-	100.00

M.Hc.—monoterpene hydrocarbons; O.M.—oxygenated monoterpenes; Phe.P.—phenylpropanoids; S.Hc.—sesquiterpene hydrocarbons.

**Table 2:** Changes in sensory scores (points) of brook trout samples during refrigerated storage.

Attributes	Treatment	Storage Time (Days)					
		0	3	6	9	12	15
Color discoloration	Control	5.0 ± 0.0 ^aA^	5.0 ± 0.0 ^aA^	5.0 ± 0.0 ^aA^	3.0 ± 0.0 ^cB^	1.6 ± 0.548 ^bC^	1.0 ± 0.0 ^cD^
	WPIf	5.0 ± 0.0 ^aA^	5.0 ± 0.0 ^aA^	5.0 ± 0.0 ^aA^	4.0 ± 0.0 ^bB^	2.6 ± 0.548 ^aC^	2.0 ± 0.0 ^bD^
	WPIf+2.5%TEO	5.0 ± 0.0 ^aA^	5.0 ± 0.0 ^aA^	5.0 ± 0.0 ^aA^	5.0 ± 0.0 ^aA^	3.0 ± 0.0 ^aB^	2.6 ± 0.548 ^aB^
Odor	Control	5.0 ± 0.0 ^aA^	5.0 ± 0.0 ^aA^	5.0 ± 0.0 ^aA^	3.0 ± 0.0 ^bB^	2.6 ± 0.548 ^aB^	1.0 ± 0.0 ^bC^
	WPIf	5.0 ± 0.0 ^aA^	5.0 ± 0.0 ^aA^	5.0 ± 0.0 ^aA^	4.0 ± 0.0 ^aB^	3.0 ± 0.0 ^aC^	2.0 ± 0.0 ^aD^
	WPIf+2.5%TEO	5.0 ± 0.0 ^aA^	5.0 ± 0.0 ^aA^	5.0 ± 0.0 ^aA^	4.0 ± 0.0 ^aB^	3.0 ± 0.0 ^aC^	2.0 ± 0.0 ^aD^
Overall acceptability	Control	5.0 ± 0.0 ^aA^	5.0 ± 0.0 ^aA^	5.0 ± 0.0 ^aA^	3.0 ± 0.0 ^bB^	1.8 ± 0.447 ^bC^	1.0 ± 0.0 ^bD^
	WPIf	5.0 ± 0.0 ^aA^	5.0 ± 0.0 ^aA^	5.0 ± 0.0 ^aA^	4.0 ± 0.0 ^aB^	2.6 ± 0.548 ^aC^	2.0 ± 0.0 ^aD^
	WPIf+2.5%TEO	5.0 ± 0.0 ^aA^	5.0 ± 0.0 ^aA^	5.0 ± 0.0 ^aA^	4.0 ± 0.0 ^aB^	3.0 ± 0.0 ^aC^	2.0 ± 0.0 ^aD^

Control-uncovered fish samples; WPIf-fish samples covered with WPI-based films; WPIf+2.5%TEO-fish samples covered with whey protein isolate-based films incorporated with 2.5% tarragon essential oil. Values are expressed as mean ± standard deviation of five responses. Means that do not share a letter (lowercase letters on column of each attribute and uppercase letters on row) are significantly different.

**Table 3:** Shelf-lives of brook trout samples at refrigerated storage.

Treatment	pH ^a,d^	TVC ^b,d^	Color Discoloration ^c,d^	Odor ^c,d^	Overall Acceptability ^c,d^
Control	6	6	6	6	6
WPIf	6	9	9	9	9
WPIf+2.5%TEO	9	9	9	9	9

Control-uncovered fish samples; WPIf-fish samples covered with WPI-based films; WPIf+2.5%TEO-fish samples covered with whey protein isolate-based films incorporated with 2.5% tarragon essential oil. ^a^ Based on a maximum permitted value of 6.2 for pH. ^b^ Based on a maximum permitted value of 6.0 log_10_ CFU/g for TVC. ^c^ Based on a minimum permitted value of 4 for the acceptance score. ^d^ Data obtained from Figure 1 and Figure 6 and Table 2, respectively.

## Data Availability

Not applicable.
